# Microstructural Properties of Cement Paste and Mortar Modified by Low Cost Nanoplatelets Sourced from Natural Materials

**DOI:** 10.3390/ma11050783

**Published:** 2018-05-11

**Authors:** Piao Huang, Liming Lv, Wei Liao, Chunhua Lu, Zhongzi Xu

**Affiliations:** 1 State Key Laboratory of Materials-Oriented Chemical Engineering, College of Materials Science and Engineering, Nanjing Tech University, Nanjing 210009, China; huangpiao@njtech.edu.cn (P.H.); liminglv@njtech.edu.cn (L.L.); lw2015@njtech.edu.cn (W.L.); zzxu@njtech.edu.cn (Z.X.); 2 Jiangsu National Synergetic Innovation Center for Advanced Materials (SICAM), Nanjing Tech University, Nanjing 210009, China

**Keywords:** CaCO_3_ nanoplatelets (CCNPs), cement-based materials, pore structure, chloride ion permeability, compressive strength

## Abstract

Nanomaterials have been widely used in cement-based materials. Graphene has excellent properties for improving the durability of cement-based materials. Given its high production budget, it has limited its wide potential for application in the field of engineering. Hence, it is very meaningful to obtain low cost nanoplatelets from natural materials that can replace graphene nanoplatelets (GNPs) The purpose of this paper is to improve the resistance to chloride ion penetration by optimizing the pore structure of cement-based materials, and another point is to reduce investment costs. The results illustrated that low cost CaCO_3_ nanoplatelets (CCNPs) were successfully obtained under alkali treatment of seashell powder, and the chloride ion permeability of cement-based materials significantly decreased by 15.7% compared to that of the control samples when CCNPs were incorporated. Furthermore, the compressive strength of cement pastes at the age of 28 days increased by 37.9% than that of the plain sample. Improvement of performance of cement-based materials can be partly attributed to the refinement of the pore structure. In addition, AFM was employed to characterize the nanoplatelet thickness of CCNPs and the pore structures of the cement-based composites were analyzed by MIP, respectively. CCNPs composite cement best performance could lay the foundation for further study of the durability of cement-based materials and the application of decontaminated seashells.

## 1. Introduction

The progress of nanotechnology has brought new insights to improve the properties of cement-based materials with the aid of nanomaterials in recent years [1,2,3]. 

Nanomaterials [4] are divided into three categories according to dimension. These three categories are 0D material, nano-silica (nano-SiO_2_) [5,6,7,8,9], nano-aluminium oxide (nano-Al_2_O_3_) [10,11,12,13,14], nano-titanium oxide (nano-TiO_2_) [15,16,17,18]; 1D material, carbon nanotubes (CNTs) [19,20,21,22,23,24,25,26]; and 2D material, graphene nanoplatelets (GNPs) [27,28,29,30,31,32,33], etc.

The durability exists an important performance of cement-based material. In the field of marine engineering, there are many factors that affect the durability of cement-based materials, including alkali-aggregate reaction, acid erosion, alkali erosion, and chloride ion penetration etc. Yet, the penetration of chloride ions will cause serious and irreversible damage to cement-based materials. Therefore, it is of great significance to improve the impermeability of cement-based materials.

GNPs is a kind of nanoscale materials consisting single-layer or multi-layer graphene flakes ranging in thickness from 1 to 100 nm. Compton [34] studied the excellent performance of crumpled GNPs in aspect of barrier light and oxygen owing to the increased tortuous path. Sullivan [35] observed the performance of GNPs composites, which demonstrated excellent mechanical strength and barrier properties. It was reported that, with the addition of GNPs, the penetration depth of water and chloride ion in cement mortar decreased significantly compared to that of the control specimens, which was attributed to the barrier properties of GNPs [36]. Meanwhile, GNPs could refine the pore structure of the cement mortar due to the distinctive layered structure and barrier property. It was found that the GNP nanocomposites were beneficial for promoting mechanical properties and impermeability in cement composites [37]. Mohammed [38] studied the mechanical properties and durability of cement-based materials incorporated with graphene oxide. The results illustrated that when the content of graphene oxide is at a very low dose, it can effectively prevent the ingress of chloride ion penetration in cement-based materials due to its barrier effect. Although great progress has been made in nanotechnology, the investment cost of GNPs is still much higher than that of traditional building materials, which limits the extensive application of nanomaterials in cement to some degree. Given the brilliant barrier effect of GNPs on chloride ion permeation in cement-based materials and its high production budget, it is very meaningful to obtain low cost nanoplatelets from natural materials that can replace GNPs.

Seashells are heavily abandoned in coastal areas [39,40,41], which are assembled from traditional ‘brick and mortar’ structure arrangements [42]. Furthermore, seashells are composite of more than 90% calcium carbonate and a small amount of organic matter [43], which means calcium carbonate nanoplatelets (CCNPs) and organic matter constitute the integrated structure of the seashells [41]. Taking into the similarity of the micromorphology between GNPs and CCNPs account, it is conceivable that CCNPs can be obtained by splitting the ‘brick and mortar’ arrangement structure of the seashells.

In the paper, low cost CCNPs were firstly obtained under alkali treatment of seashells powder. Moreover, the effect of CCNPs incorporated on the performance of cement-based materials was observed. The nanoscale thickness of CCNPs was characterized by atomic force microscope (AFM) images. Besides, the pore structure of cement composites was characterized by mercury intrusion porosimetries (MIP) and the mechanism of the introduction of CCNPs was revealed. The best performance of CCNP composite cement lays the foundation for further study of the durability of cement-based materials and the application of decontaminated seashells.

## 2. Materials and Methods 

### 2.1. Materials

Type I Portland Cement (CEM I 42.5) plays the role of the main binder, which was provided by Qufu China United Cement Co., Ltd. (Qufu, China). Mussel shells were collected from Lianyungang City, Jiangsu province, which were arbitrarily abandoned in the seafood market. Sodium hydroxide (AR) and ethanol solution (AR) were purchased from Xilong Reagent (Shantou, China).

### 2.2. Acquisition of CCNPs 

Mussel seashells were rinsed and dried in an oven at 65 ± 5 °C for 24 h, followed by cracking and grounding into powders to pass 75 μm sieve by using a vibration crusher. Sodium hydroxide solution with a mass fraction of 5% was used as the reaction solvent, accompanied by water-curing treatment at 80 °C with maintaining magnetic stirring for 7 h, then ultrasonic dispersion for 1.5 h and centrifugal cleaning solution to neutral, finally the powder was dried in an oven at 65 °C.

### 2.3. Mix Composition

Cement paste was mixed with water-cement at mass ratio of 0.28, which meets the fluidity of cement paste under the standard requirements. 0.01, 0.02, 0.03, and 0.04% of CCNPs by weight of cement were used in this research.

Cement mortar was mixed with cement to sand at mass ratio of 1:3. The water to cement ratio of 0.5. 0.01, 0.02, 0.03, 0.04, 0.07, and 0.1% CCNPs by weight of cement were used in this study.

### 2.4. Test Methods

The chemical composition of mussel seashells powder was determined by X-ray fluorescence spectrometry (XRF, ARL ADVANT’XP). In addition, the particle size distribution of the mussel shell powder was measured by using a laser particle size analyzer (Malvern, Mastersizer 2000). Rigaku D/Max-2500 XRD, Cu-Kα (1.541874 Å) was used to investigate the phase structure of the prepared samples.

The physical properties of CCNPs were tested by the following characterization: particle morphology disclosed through a scanning electron microscope (SEM, JEOL JSM-5900); and the thickness of CCNPs were considered by atomic force microscope (AFM, Veeco Autoprobe CP Research).

### 2.5. Mechanical Test

The compressive strength of cement pastes was examined at various ages of 3, 7, 28 and 56 days using 2 cm cube specimens. A mean value of six paste cubes was used for the compressive strength test. The compressive strength of cement mortar was examined at various ages of 3, 7, 28 days by testing 4 cm cube specimens. A mean value of six mortar cubes was used for the compressive strength test.

### 2.6. Accelerated Chloride Penetration Test

**Figure 1:**
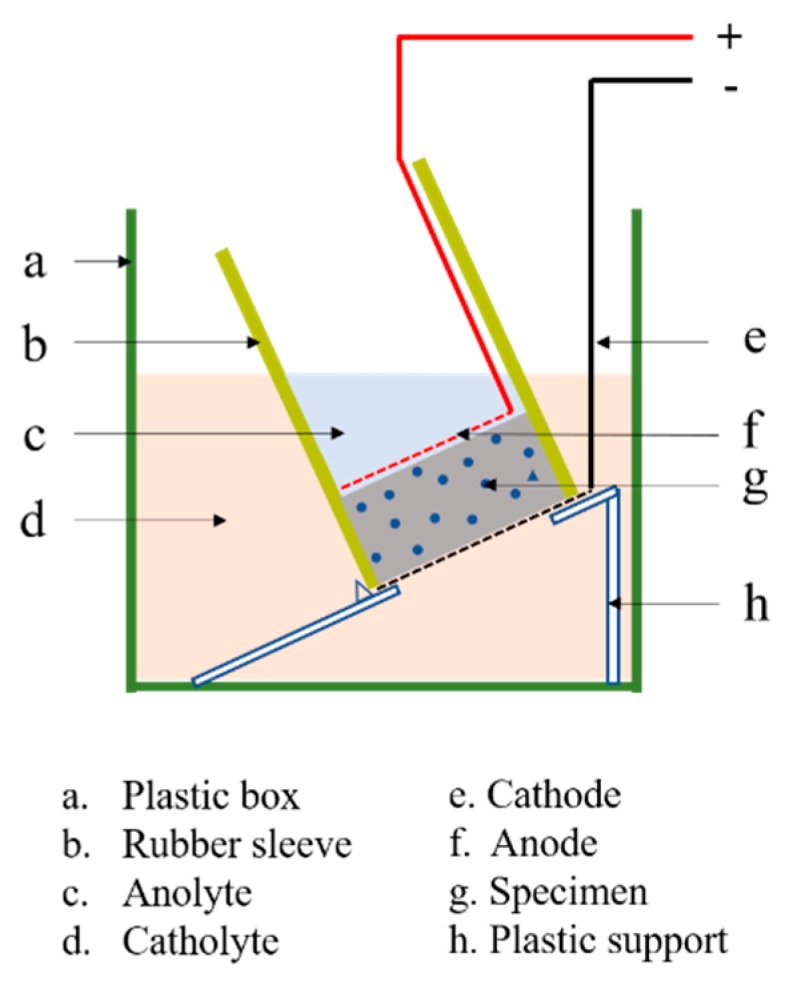
Arrangement of the migration set-up.

NT build 492 [44] (RCM method) was applied to test the chloride diffusion coefficient of cement mortar samples as shown in Figure 1. The principle is used by the effect of an applied electric field to move the chlorine ions outside the specimen to the inside of the specimen. The RCM method is an effective method to evaluate the resistance against chloride ion permeability of cement-based materials. The test was completed on the basis of establishing migration cell. 

In this study, specimens were obtained from Φ110 × 220 mm cement mortar cylinders. The migration cell is full of 0.1 M/L NaOH (13.33 g L^−1^), and 10 wt % NaCl solution. Besides, because of the apparent diffusion coefficient of chloride ion in cement-based materials, the other half of the specimen was spurted with AgNO_3_ solution to disclose the chloride penetration depth, which can be quickly and accurately measured by silver nitrate colorimetric method.

The calculation of the chloride penetration value from migration test was obtained with the simplified Equation (1) [41]

(1) $$D_{RCM}=\frac{0.0239*(273+T)*L}{(U-2)*t}*(X_{d}-0.0238*\sqrt{\frac{((273+T)*L*X_{d}}{U-2}})$$

where D*_RCM_* is the chloride ion transfer coefficient of unsteady chlorine ion, accurately to 0.1 × 10^−2^ m^2^/s. *U* is the absolute value of the voltage, units by volt (V). *L* is the thickness of the specimen, accurately to 0.1 mm. *T* is the mean value of the initial temperature and the end temperature of the anodic solution. *X*_d_ is the mean value of the penetration depth of the chloride ion, accurately to 0.1 mm. The half of the specimen was measured for 10 values. *T* is the duration time of the test.

## 3. Results and Discussion

### 3.1. Properties of CCNPs

**Figure 2:**
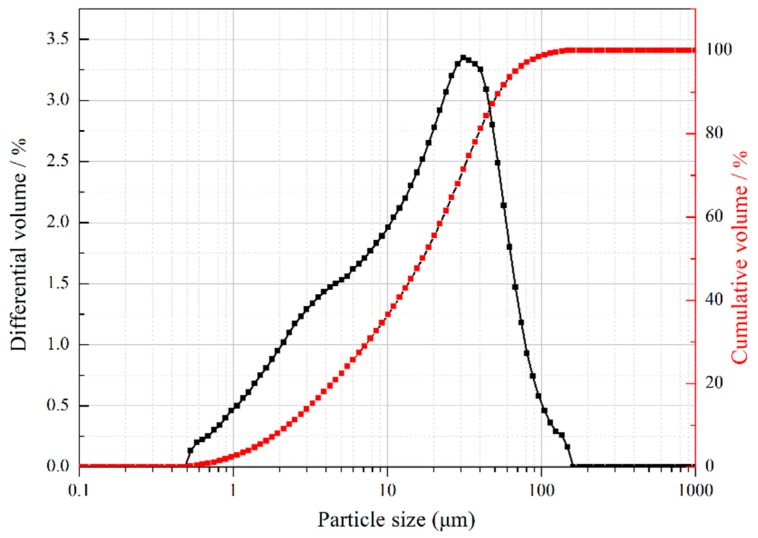
Particle size distribution of mussel shells powder.

It can be seen from the Figure 2 that the median particle size of mussel shell powder is 15.9 μm. The particle size of mussel shell powder is between 0.5–200 μm.

The chemical composition of mussel seashells was given in Table 1. It can be noted that mussel shells are absolutely made up of inorganic CaCO_3_ phases (approximately 96.5%) and minor mineral composition. The mineral phase of calcium carbonate is demonstrated to be calcite or aragonite [39,41,43]. In addition, shells also contain small amounts of organic components, which are closely related to the growth of the shells. In mussel shells, the CaO composition is found to be 54.03% with a high loss on ignition (LOI) value of 44.48%. The LOI is owing to the decomposition of CaCO_3_.

**Figure 3:**
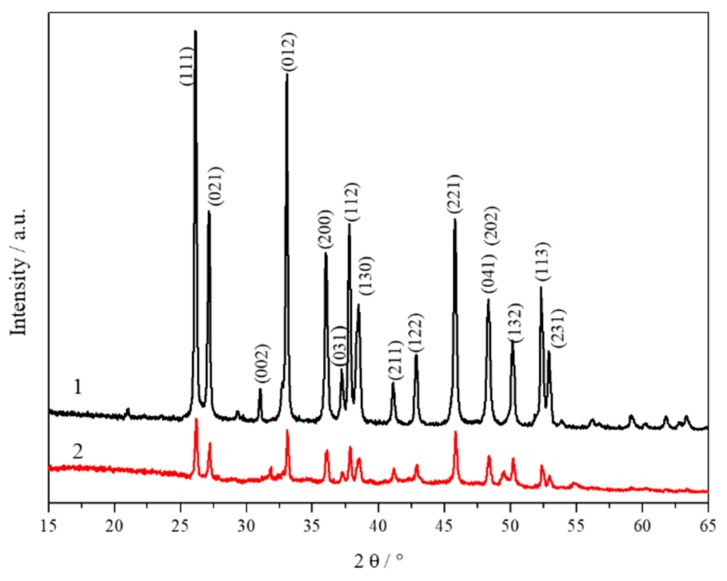
X-ray diffraction patterns (1) CCNPs; (2) mussel shells powder.

XRD was used to investigate the phase structure of the prepared samples. Figure 3 shows the XRD patterns of the CCNPs and mussel shells powder, showing the diffraction peaks of the prepared CCNPs and mussel powder, corresponding to the (111), (021), (002), (012), (200), (031), (112), (130), (211), (122), (221), (041), (132), (113), and (231) planes, indicating that the pure CNNPs were prepared. However, it is worth noting that the peak strength of CCNPS is stronger than that of mussel powder, and CCNPS can be stripped from the original mussel powder in all directions.

**Figure 4:**
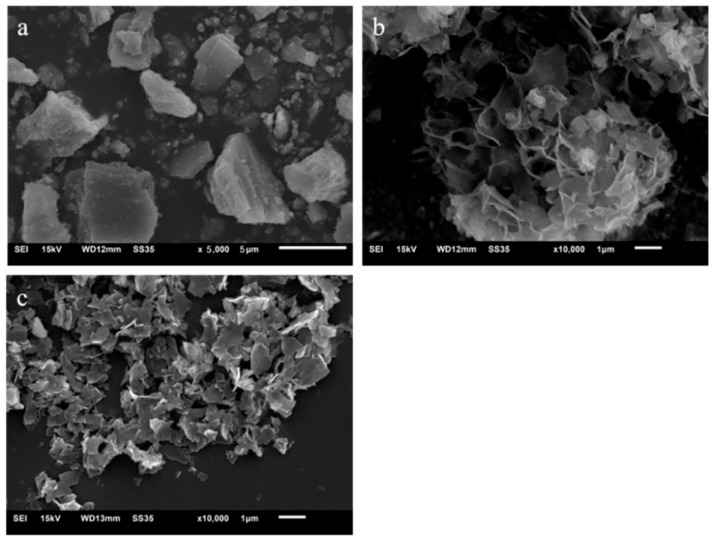
Scanning electron images. (**a**) mussel shells powder; (**b**) CCNPs-1; (**c**) CCNPs-2.

Mussel shells have traditional ‘brick and mortar’ arrangement on the fractured surfaces of the mussel shells [39], which is an organic whole consisting of calcium carbonate aragonite tablets and organic matter. Hence, it serves as a basis for the experimental process in which mussel shells can be processed into platelets. Figure 4 shows the micro morphology of mussel shells powder and CCNPs. Referring to Figure 4a, main size of pristine mussel shells power is about 10 μm. Figure 4b,c shows the morphology of CCNPs obtained after treatment of quail shell powder with 5% NaOH solution under water bath treatment conditions. According to Figure 4b, under alkaline conditions, 5% sodium hydroxide solution has a good deproteinization effect on the mussel shells powder [45]. Figure 4c presents microscopic morphology of CCNPs with deciduous shape, and the small unit of CCNPs is well dispersed. As shown in Figure 4b, CCNPs still have large morphological difference but relatively uniform size of 1 μm. It can be observed that the size of CCNPs is obviously smaller than that of mussel shell powder, but their thicknesses cannot be precisely observed from the SEM images. 

**Figure 5:**
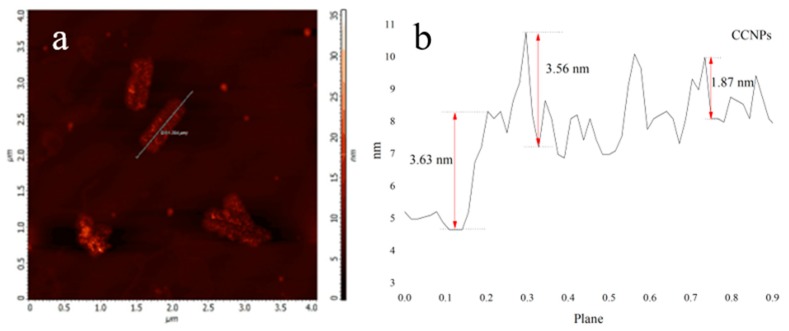
High magnification AFM diagram of CCNPs. (**a**) Morphology; (**b**) height.

High magnification AFM was utilized to analyses the thickness and the finer structure of CCNPs, which was presented in Figure 5. Compared with the mussel shell powder, the size of CCNPs is smaller and the thickness is obviously reduced. As can be seen from Figure 5a, show the flake shape morphology of CCNPs, which is consistent with the results in Figure 4c. At the same time, Figure 5b illustrates the height of CCNPs (namely its thickness) ranges from 1 nm to 4 nm. Compared with the particle size of mussel shell powder in Figure 2, the particle size of CCNPs decreased from about 16 to 1 μm. Combined with SEM and AFM analysis, the result obviously reveals that CCNPs could be successfully prepared by using alkali treatment assisted with ultrasonic.

### 3.2. Properties of Cement-Based Materials

#### 3.2.1. Pore Size Distribution

The porosity and pore distribution are two main factors affecting the durability and strength of cement-based materials. Cementitious materials with lower porosity and finer pores show better durability and higher strength. The effect of the incorporation of CCNPs on the porosity and pore size distribution was determined by MIP is discussed below.

**Figure 6:**
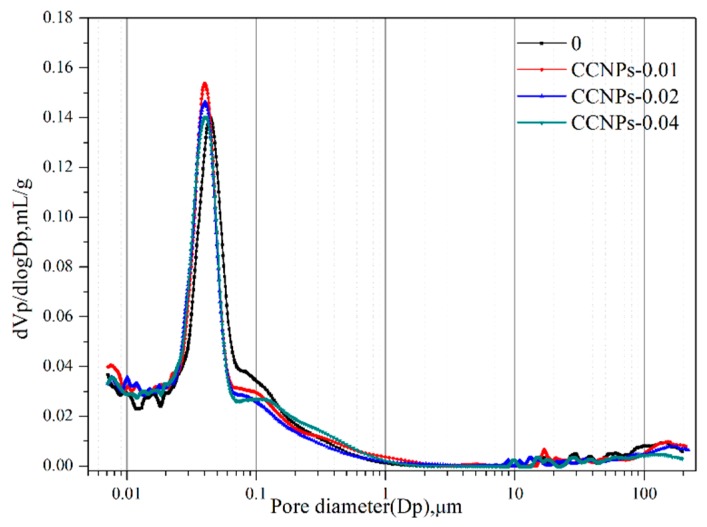
Pore size Distribution of CCNPs composite cement pastes.

Figure 6 illustrates the pore size distribution of CCNPs composite cement pastes. Based on the MIP test, after 28 days curing, Table 2 shows the total porosity of 0, CCNPs-0.01, CCNPs-0.02, and CCNPs-0.04 when CCNPs account for 0, 0.01, 0.02, and 0.04% of the weight of the cement. According to Table 2, the results illustrate the total porosities are 15.59, 15.42, 15.51, and 15.58%, correspondingly. Apparently, there are no significant changes in the total porosity, but pore size distribution is affected a lot by the incorporation of CCNPs. For CCNPs-0.01, the harmless pore (<20 nm) increased from 18.54% to 22.97%, and the less harm pore (20–50 nm) increased from 37.91% to 47.52%. In addition, the performance displays that it could optimize the pore structure of cement-based materials with a little amount of CCNPs in the cement mix, which could be attributed to the flake structure of CCNPs (refer to Figure 4c). CCNPs are nanometer scale, with the thickness of several nanometers, which easily generate sheet wrinkle and interlock with some more sheets. Moreover, CCNPs are hydrophilic material, it could be believed that nanoscale CCNPs can lead to the ordinary influence by optimization of pore structure in cement-based materials [2,3,4].

It can also be observed that with the 0.04% of CCNPs incorporating to cement mortar, the result shows that both the less harm pore and large pore increase while the harmless pore decreases compared with the control sample. That is because when CCNPs are introduced into the cement, they will be surrounded by the hydration product of the cement and remain in it. When chloride ions intrude into cement, the permeation path of chloride ion can be changed and extended, thus improving its impermeability [5].

However, with the increasing content of CCNPs, the properties of cement-based materials decrease even lower than those of control samples. It could be concluded that, due to the effect of CCNPs stacking effect and its irregular shapes, coupled with the combined effect of weakening the nanoscale effect, when the amount of CCNPs increases, more water was needed for the dispersion of CCNPs, which easily results in an aggregation effect in the mix. As reported [40,41,42,43,44,45], the pore structure of cement-based materials influences its physical properties directly, including compressive strength and durability. 

#### 3.2.2. Chloride Penetration Test

**Figure 7:**
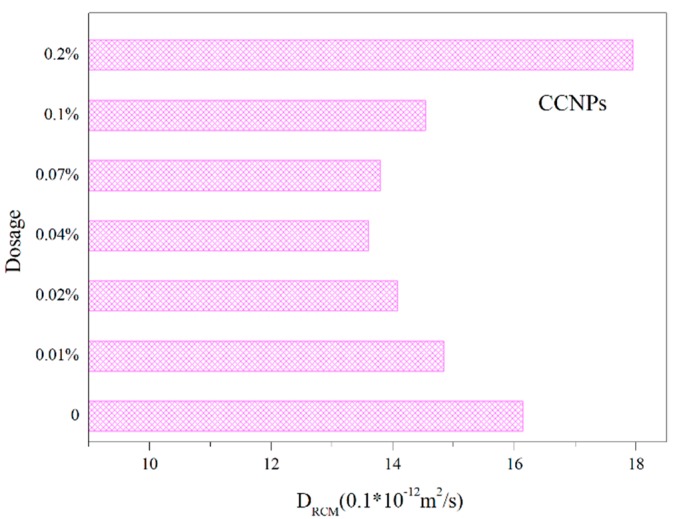
Chloride depth penetration results of cement mortar specimens with different amounts of CCNPs.

The result of the chloride penetration impermeability of cement mortar specimens is shown in Figure 7. It could be concluded that the incorporation of CCNPs can effectively affect the permeability of chloride in cement mortar. When the amount of CCNPs increased from 0.01% to 0.04%, the chloride penetration impermeability of the cement mortar is getting lower, that is, permeability resistance of cement mortar improves. Furthermore, cement mortar with 0.04% of CCNPs incorporated exhibits the lowest chloride depth penetration, reduced by 15.7% than that of the control samples. However, when the content of CCNPs is higher than 0.04%, the permeability coefficient of the cement mortar will increase, but it is still lower than that of the control samples. Until the content of CCNPs reaches to 0.2%, the permeability coefficient of the mortar is higher than that of the control samples. In short, a small amount of CCNPs plays an important role in optimizing the permeability of cement mortar and enhancing its durability, when the amount of CCNPs exceeds a certain value of 0.04%, the effect of the addition of CCNPs on the impermeability of the mortar will be weakened until the opposite effect occurs.

#### 3.2.3. Compressive Strength of Cement Paste and Cement Mortar

**Figure 8:**
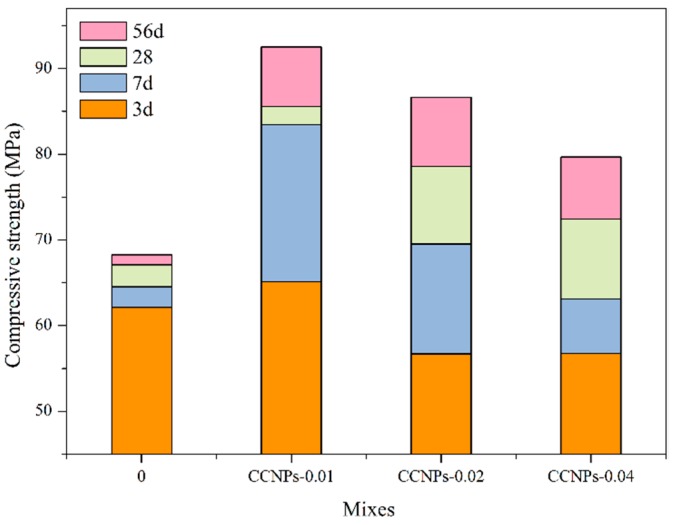
Compressive strength diagram of cement pastes with different amounts of CCNPs.

As the curing age extended, the compressive strength increased correspondingly, because the process of the cement hydration had gradually accomplished. Figure 8 shows the change of compressive strength of cement paste with respect to four different CCNPs dosage ratios of 0, 0.01, 0.02, and 0.04%. It is noted that the incorporation of CCNPs shows a significant effect on cement paste compressive strength, with value ranging from 55 to 92 MPa, accordingly. The evolution of cement hydration shows that the trend of strength changes gradually milder. Moreover, referring to Figure 8, the compressive strength achieves the highest value when incorporating 0.01% of CCNPs regardless of curing ages, and the compressive strength increased by 37.9% than that of the control samples, respectively. However, when the amount of CCNPs increases to 0.04%, increment of compressive strength is lower than that of 0.01%. In general, the compressive strength increases with the CCNP dosage.

**Figure 9:**
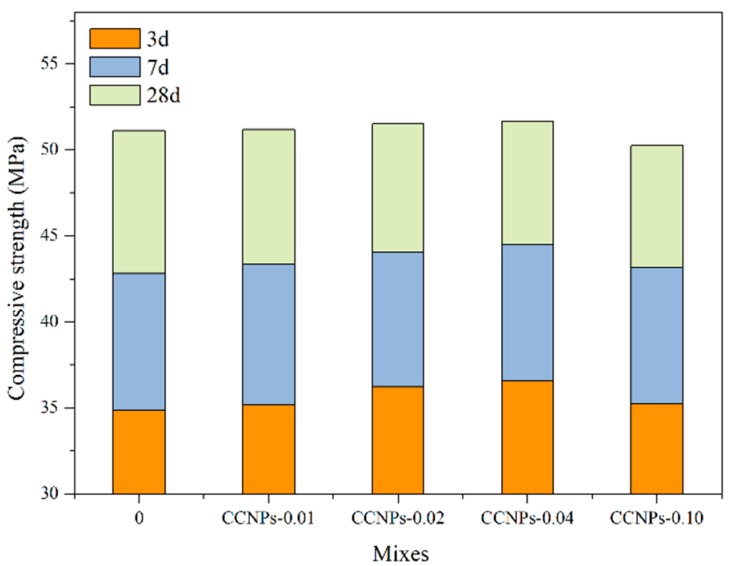
Compressive strength of cement mortar with different amounts of CCNPs.

Figure 9 shows the change in compressive strength of cement mortar with 0, 0.01, 0.02, and 0.04% for four different CCNPs dose ratios. With the extension of curing time, the compressive strength of cement mortar is gradually increased. It can be observed that when the CCNPs are incorporated to cement mortar, there was no significant change in compressive strength at 28 days compared to the control sample. The reason for this phenomenon is that a large amount of sand is added to the cement mortar, which greatly dilutes the content of CCNPs in the cement, thereby weakening the effect of CCNPs on the mechanical properties of the cement paste. However, at the curing time of 3 days, the compressive strength of the cement mortar increased with the increase of the amount of CCNPs added, indicating that the CCNPs play a nucleating role in the cement mortar and promote the hydration of the cement.

## 4. Conclusions

Seashell landfill brings about a great deal of pollution and waste into the environment. Recycling waste seashells can effectively solve the problems. In this study, Nanoscale CCNPs were obtained from alkali-treated mussel shells powder. With incorporating CCNPs to the cement-based materials at lower dosage, the results indicate the positive effect on the resistance of chloride ion penetration and the compressive strength. Besides, it can bring considerable economic and environmental efficiency. Therefore, the following conclusions can be made:

Nanoscale CCNPs can be successfully obtained from mussel shell powder by alkali treatment. Specific test process: the mussel shell powder was placed in the mass fraction of 5% sodium hydroxide solution, 80 °C water bath heating, and supplemented by 1.5 h of ultrasound correspondingly.

CCNPs have an efficacious influence on chloride ion impermeability and compressive strength by refinement of the pore structure of cement-based materials. The cement mortar incorporating 0.04% CCNPs resulted in a 16.1% reduction in chloride penetration as compared with the control mortar. Also, the cement paste including 0.01% CCNPs provided 37.9% higher compressive strength than the control paste. Yet, CCNPs should remain in the low dosage because of the agglomeration effect.

## Figures and Tables

**Table 1:** Chemical composition of mussel shells powder and CEM I 42.5 Portland cement.

Chemical Composition (%)	Cement	Mussel Shells Powder
CaO/CaCO_3_	54.03/96.48	63.82
Na_2_O	0.72	0.11
SiO_2_	0.23	21.02
SO_3_	0.13	-
Al_2_O_3_	0.06	5.21
MgO	0.04	2.56
Fe_2_O_3_	0.04	3.4
P_2_O_5_	0.03	-
K_2_O	0.01	0.68
LOI	44.48	2.95

**Table 2:** Porosity and pore size distribution of different mixes.

Samples	Total Intruded Vol. (mL/g)	Total Porosity (%)	Pore Size Distribution/%			
			<20 nm	20–50 nm	50–200 nm	>200 nm
0	0.0804	15.59	18.54	37.91	29.76	13.79
CCNPs-0.01	0.0713	15.42	22.97	47.52	17.38	12.13
CCNPs-0.02	0.0767	15.51	20.57	44.68	21.92	12.77
CCNPs-0.04	0.0786	15.58	17.84	44.29	23.43	21.82
